# The Level of Preoperative Plasma KRAS Mutations and CEA Predict Survival of Patients Undergoing Surgery for Colorectal Cancer Liver Metastases

**DOI:** 10.3390/cancers12092434

**Published:** 2020-08-27

**Authors:** Jiri Polivka, Jindra Windrichova, Martin Pesta, Katerina Houfkova, Hana Rezackova, Tereza Macanova, Ondrej Vycital, Radek Kucera, David Slouka, Ondrej Topolcan

**Affiliations:** 1 Department of Histology and Embryology and Biomedical Center, Charles University, Faculty of Medicine in Pilsen, Karlovarska 48, 30166 Pilsen, Czech Republic; jiri.polivka2@lfp.cuni.cz; 2 Laboratory of Immunoanalysis, University Hospital in Pilsen, E. Benese 13, 30599 Pilsen, Czech Republic; windrichovaj@fnplzen.cz (J.W.); rezackovah@fnplzen.cz (H.R.); kucerar@fnplzen.cz (R.K.); slouka@fnplzen.cz (D.S.); topolcan@fnplzen.cz (O.T.); 3 Department of Biology, Charles University, Faculty of Medicine in Pilsen, alej Svobody 76, 32300 Pilsen, Czech Republic; katerina.houfkova@lfp.cuni.cz (K.H.); tereza.macanova@lfp.cuni.cz (T.M.); 4 Department of Surgery, University Hospital in Pilsen, E. Beneše 13, 30599 Pilsen, Czech Republic; vycitalo@fnplzen.cz

**Keywords:** colorectal cancer, liver metastasis, circulating tumor DNA, cell-free DNA, ctDNA, CEA, liquid biopsy

## Abstract

Colorectal cancer (CRC) belongs to the most common cancers. The liver is a predominant site of CRC dissemination. Novel biomarkers for predicting the survival of CRC patients with liver metastases (CLM) undergoing metastasectomy are needed. We examined KRAS mutated circulating cell-free tumor DNA (ctDNA) in CLM patients as a prognostic biomarker, independently or in combination with carcinoembryonic antigen (CEA). Thereby, a total of 71 CLM were retrospectively analyzed. Seven KRAS G12/G13 mutations was analyzed by a ddPCR™ KRAS G12/G13 Screening Kit on QX200 Droplet Digital PCR System (Bio-Rad Laboratories, Hercules, CA, USA) in liver metastasis tissue and preoperative and postoperative plasma samples. CEA were determined by an ACCESS CEA assay with the UniCel DxI 800 Instrument (Beckman Coulter, Brea, CA, USA). Tissue KRAS positive liver metastases was detected in 33 of 69 patients (47.8%). Preoperative plasma samples were available in 30 patients and 11 (36.7%) were KRAS positive. The agreement between plasma- and tissue-based KRAS mutation status was 75.9% (22 in 29; kappa 0.529). Patients with high compared to low levels of preoperative plasma KRAS fractional abundance (cut-off 3.33%) experienced shorter overall survival (OS 647 vs. 1392 days, *p* = 0.003). The combination of high preoperative KRAS fractional abundance and high CEA (cut-off 3.33% and 4.9 µg/L, resp.) best predicted shorter OS (HR 13.638, 95%CI 1.567–118.725) in multivariate analysis also (OS HR 44.877, 95%CI 1.59–1266.479; covariates: extend of liver resection, biological treatment). KRAS mutations are detectable and quantifiable in preoperative plasma cell-free DNA, incompletely overlapping with tissue biopsy. KRAS mutated ctDNA is a prognostic factor for CLM patients undergoing liver metastasectomy. The best prognostic value can be reached by a combination of ctDNA and tumor marker CEA.

## 1. Introduction

Colorectal cancer (CRC) belongs to the most common cancers with more than 1.8 million new cases worldwide per year [1]. Moreover CRC accounts for 9% of all cancer-related deaths. Colorectal cancer liver metastases (CLM) are the predominant distant recurrence developing in 25–30% of CRC patients [2,3]. Liver metastasectomy provides potentially curative treatment for those affected by CLM with a five-year survival up to 47–60% [2,4]. However, recurrences occur in 40–75% of patients after liver surgery. Therefore, effective biomarkers predicting patients’ survival and disease relapse in this specific clinical scenario are urgently needed. Accurate prognosis assessment will help in deciding on an appropriate treatment or facilitate the possible inclusion of patients in any of the ongoing studies.

Once the presence of circulating acids (circulating cell-free DNA- cfDNA and non-coding RNAs) in body fluids was observed, these molecules attracted interest of cancer research. The benefit of detection of cell-free DNA in plasma or serum of cancer patients is to gain the knowledge about the presence of mutations typical for tumor tissue and so to get minimally invasive diagnostic, prognostic and predictive tool. From a clinical practice perspective, it makes sense to detect the most common mutations (circulating cell-free tumor DNA- ctDNA) for a given oncological disease (diagnostic, prognostic) or mutations that can help in the prediction of treatment.

Carcinogenic Kirsten rat sarcoma viral oncogene homolog (KRAS) is the most frequently mutated proto-oncogene in CRC. Up to 45% of CRC comprise KRAS mutations. KRAS encodes a 21 kDa a membrane-bound small GTPase and is a member of the Ras oncogene family includes also HRAS and NRAS proto-oncogenes. KRAS is located at short arm of chromosome 12 (12p12.1), spans approximately 38 kb and the most frequent mutations in this gene, point substitutions in codons 12 and 13 [5,6]. Oncoprotein KRAS aberrantly activate RAS/MAPK pathway and thus contributes to cell cycle deregulation [7].

Mutated RAS genes were the first tumor specific gene sequences detected in the blood from patients with cancer [8,9]. For the first time the presence of mutated KRAS2 sequences was detected in the blood of patients with pancreatic cancer in 1994 by Sorenson et al. [10]. Since 2000, a number of studies have been published to test the predictive importance of mutations in the KRAS oncogene for low molecular weight inhibitor and biological therapy. Now is established that KRAS mutations together with mutations in another proto-oncogene NRAS (exons 2, 3, and 4) are predicting the lack of treatment efficacy of anti-epidermal growth factor receptor (EGFR) monoclonal antibodies (mAb) cetuximab and panitumumab [11,12,13,14]. The US and European clinical practice guidelines involve indications for RAS testing (KRAS and NRAS mutations) before the use of anti-EGFR agents [15,16]. The standard techniques evaluating KRAS mutation status for the decision about anti-EGFR mAb therapy are based on formalin-fixed, paraffin-embedded (FFPE) specimens of tumor tissue obtained during surgery (tissue biopsy). As an alternative, recently developed methods for so-called liquid biopsy analyzing circulating tumor DNA (ctDNA) from peripheral blood can provide a rapid KRAS genotyping that is relatively non-invasive and with a minimal risk of complications compared to tissue biopsy [17,18]. Studies show that blood detected mutated RAS gene bears prognostic value in primary and metastatic colorectal cancer [19,20,21,22,23,24,25,26,27,28]. At present, the possibility of identification of mutated KRAS oncogene in ctDNA is extended by determining its exact amount at very low level (for example by the ddPCR technique) and thus the relationship of the mutated KRAS levels to the clinical pathological characteristics of the disease can be examined.

Recent studies have indicated that combining multiple markers may improve the accuracy of diagnostic and assessment of the prognosis [29]. In accordance with this idea, we also combined the plasma ctDNA KRAS marker with routinely used tumor marker in gastrointestinal cancers-carcinoembryonic antigen (CEA). The carcinoembryonic antigen (CEA, CEACAM5, and CD66e) is a 180-kDa N-linked glycoprotein that is not normally produced in significant quantities after birth (<0.49 ng/mL in the blood of healthy adults) but is aberrantly over-expressed by epithelial cancers including cancers of the gastrointestinal tract, breast, lung, ovary and pancreas [30,31,32]. CEA is the prototypic member of a family of highly related cell surface glycoproteins that includes 12 carcinoembryonic antigen-related cell adhesion molecules (CEACAMs). CEA and CEACAM-1 are integral components of the apical glycocalyx human colonic epithelium [33]. However, a mechanistic role for soluble CEA in tumor progression and metastasis remains to be established [32]. CEA is the biochemical gold standard for early detection of cancer recurrence, recommended by both the American Society of Clinical Oncology (ASCO) and the European Group on Tumor Markers (EGTM) [34]. This marker is also one of the most commonly used prognostic factors for CRC [35]. However, the sensitivity of CEA is not considered to be sufficient [36]. Plasma concentration is not consistently elevated in colorectal cancer and may be undetectable or present at only low levels with poorly differentiated tumors [37]. At the most commonly reported CEA threshold of 5 μg/L shows to detect colorectal cancer recurrence the sensitivity 71% and the specificity 88% [38].

In this study, we evaluated the concordance and prognostic value KRAS mutations in ctDNA-based liquid biopsy compared to FFPE-based tissue biopsy of primary CRC and corresponding CLM assessed by droplet digital PCR methodic (ddPCR). Therefore, to establish the possibility of using plasma levels of mutated KRAS ctDNA as a supplement or replacement for FFPE tissue (biopsies) for prediction of treatment in cases where tissue is not available. We assessed the possibility for the quantification of KRAS mutant alleles in ctDNA to serve as an independent prognostic factor for patients undergoing surgery for liver metastases. The possible synergic prognostic value of combination of KRAS ctDNA-based liquid biopsy with the conventional CRC biomarker-carcinoembryonic antigen (CEA) was also evaluated.

## 2. Results

### 2.1. The Presence and the Level of KRAS Mutations in Tissue of Primary Tumor and Liver Metastases

KRAS status in the tumor tissue of primary colorectal cancer (CRC) was available in 63 pts. Tissue KRAS positivity (tKRAS+) was detected in 33 of 63 cases (52.4%). The median tissue KRAS fractional abundance (tFA; proportion of the mutant allele in FFPE total DNA) was 15.66% ranging from minimum 0% up to maximum 49.31%. In colorectal cancer liver metastases (CLM), KRAS status was available in 69 patients. tKRAS+ was found in 33 of 69 cases (47.8%). The median tFA for all patients was 0% ranging from minimum 0% up to maximum 79.95%. The median tFA for 33 KRAS positive patients only was 27.3% (minimum 11.06%, maximum 49.31%).

The overall percentage agreement between primary CRC and CLM tissue KRAS mutation status (i.e., positive or negative) was 93.4% (57/61; kappa, 0.529 (*p* = 0.002)). In contrast, four samples (6.6%) experienced discordant status (Table 1). The KRAS fraction abundance positively correlated between primary tumor tissue and liver metastases samples (R = 0.8, *p* < 0.001, *n* = 61).

### 2.2. The Presence and the Level of KRAS Mutations in Plasma

The plasma samples obtained before primary CRC surgery were available in 7 patients. Plasma KRAS positivity (pKRAS+) was detected in three of seven samples (42.9%). The median plasma KRAS fractional abundance (pFA) was 0% ranging from minimum 0% up to maximum 42.26%. The median pFA for three pKRAS+ patients only was 1.37%. The corresponding plasma samples after CRC surgery were available for four patients. For three of them, negative preoperative pKRAS remained negative after the surgery as well. One patient experienced decrease of pFA following primary tumor resection (from 42.26% preoperative to 22.85% postoperative, resp.). Both tissue and preoperative plasma samples were available in seven patients. The concordant KRAS status in CRC tissue and plasma samples was found in six of seven patients (85.7%), whereas one patient (14.3%) experienced discordant status.

Before surgery for colorectal cancer liver metastases, preoperative plasma samples were available in 30 patients. Eleven (36.7%) of them were plasma KRAS positive (pKRAS+). The overall percentage agreement of preoperative plasma KRAS status (i.e., positive or negative) between CRC and CLM in the same patient was 80% (4 in 5; kappa, 0.545 (*p* = 0.171)). The median pFA was 0% ranging from minimum 0% up to maximum 48%. The median pFA for 11 pKRAS+ patients only was 3.33% (min. 1.35%, max. 48%). The KRAS fraction abundance positively correlated between preoperative plasma samples and tissue samples of liver metastases (R = 0.649, *p* < 0.001, *n* = 29). Preoperative KRAS fraction abundance did not correlate with a number of liver metastases (R = 0.117, *p* = 0.536) or the extent of liver metastases (R = −0.17, *p* = 0.37).

**Figure 1:**
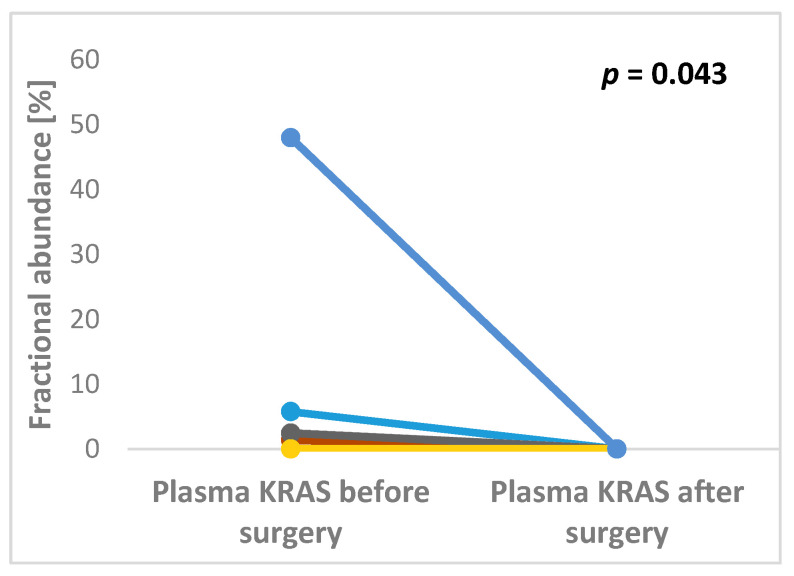
Significant changes in KRAS fractional abundance between pre- and post-operative plasma samples in patients undergoing surgery for colorectal cancer liver metastases (*p* = 0.043).

Both pre- and post-operative plasma samples were available in 17 pts. Of them, 12 preoperative pKRAS negative remained negative after surgery, whereas five preoperative pKRAS+ became negative. Therefore, all patients with available plasma samples were pKRAS negative after liver surgery (Figure 1).

Both tissue and plasma samples were available for 29 pts. Of them, pKRAS+ was detected in 10 of 16 tKRAS+ patients (62.5%), whereas 12 of 13 tKRAS negative patients were pKRAS negative (92.3%) (Table 2). The overall percentage agreement between plasma-based and tissue-based KRAS mutation status was 75.9% (22 in 29; kappa, 0.529 (*p* = 0.002)).

### 2.3. The Preoperative and Postoperative Serum Level of CEA in Patients Undergoing Liver Surgery

**Figure 2:**
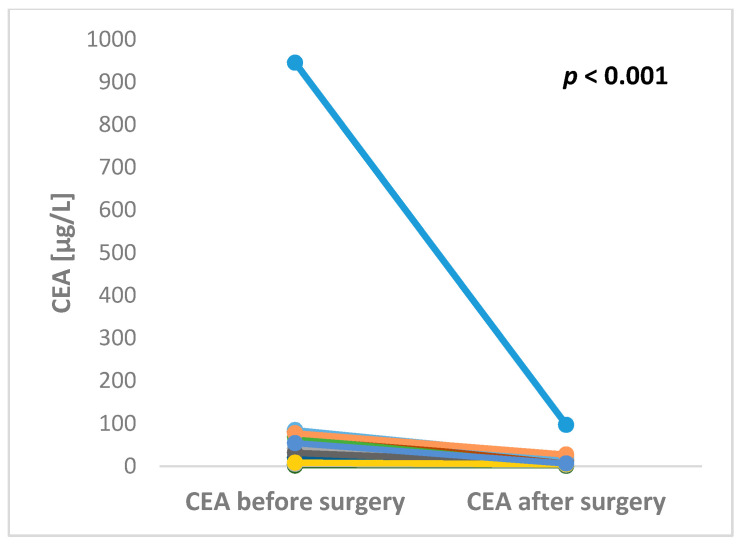
Significant changes in CEA levels between pre- and post-operative blood samples in patients undergoing surgery for colorectal cancer liver metastases (*p* < 0.001).

The preoperative serum level of CEA was available in 29 patients. The median preoperative CEA level was 11.9 µg/L ranging from minimum 0.8 up to maximum 945.4 µg/L. CEA after liver surgery was available in 17 patients. The median postoperative CEA level was 4.2 µg/L ranging from minimum 0.5 up to maximum 96.5 µg/L. Both pre- and postoperative levels of CEA were available in 16 patients. All of these patients experienced decrease of CEA level after liver surgery with the maximum fold change 43.3 (Figure 2).

### 2.4. Survival Analysis

The median distant metastasis free survival (DMFS) after the surgery of primary tumor was 210 days. The median disease-free survival (DFS) and overall survival (OS) after liver surgery was 423 and 1269 days, respectively. Both the patients with KRAS positive vs. KRAS negative primary tumor tissue experienced similar DMFS (median 210 vs. 186 days, *p* = 0.215). Similarly, there were no differences in DFS (median 475 vs. 357 days, *p* = 0.245) and OS (median 1368 vs. 1230 days, *p* = 0.783) after liver surgery between patients with KRAS positive and negative liver metastasis. The percentage increment of KRAS fractional abundance in primary tumor tissue (tFA) did not influence patients´ DMFS (HR 0.989, *p* = 0.185). Similarly, liver metastasis KRAS tFA did not influence DFS (HR = 1, *p* = 0.962) or OS (HR = 1, *p* = 0.968) after liver surgery. The analysis of the prognostic impact of the other clinicopathological factors showed that patients´ DFS was not affected by tumor grade (grade 2 vs. 1 HR = 1.099, *p* = 0.78; grade 3 vs. 1 HR = 0.737, *p* = 0.593), number of liver metastases (≥2 vs. 1 HR = 1.378, *p* = 0.209), extent of liver metastases (increment in size HR = 0.998, *p* = 0.727), extrahepatic disease (present vs. absent HR = 0.85, *p* = 0.756). Similarly there were no differences in OS in relation to tumor grade (grade 2 vs. 1 HR = 0.602, *p* = 0.399; grade 3 vs. 1 HR = 0.807, *p* = 0.703), number of liver metastases (≥2 vs. 1 HR = 1.184, *p* = 0.545), extent of liver metastases (increment in size HR = 1.004, *p* = 0.548), extrahepatic disease (present vs. absent HR = 1.253, *p* = 0.668).

**Figure 3:**
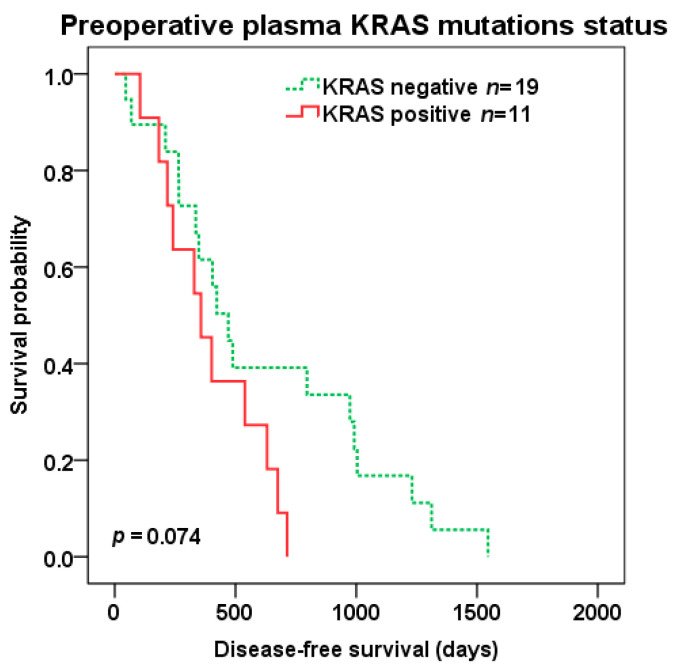
Disease-free survival (DFS) after liver surgery in patients with KRAS positive (red color) vs. negative (green color dotted line) pre-operative plasma samples.

There was a trend to shorter DFS (median 357 vs. 470 days, *p* = 0.074) (Figure 3) after liver surgery in patients with KRAS positive vs. negative preoperative plasma samples, but not OS (median 1269 vs. 1390 days, *p* = 0.234).

On contrary, percentage increment in preoperative plasma KRAS fractional abundance (pFA) predicted shorter OS (HR 1.04, *p* = 0.049) and trend to shorter DFS (HR 1.037, *p* = 0.073) after liver surgery. Importantly, preoperative pFA were independent predictor for DFS (HR 1.044, *p* = 0.041) and OS (HR 1.05, *p* = 0.021) in multivariate analysis including other covariates (also including parameters: extend of liver resection and presence of biological treatment).

**Figure 4:**
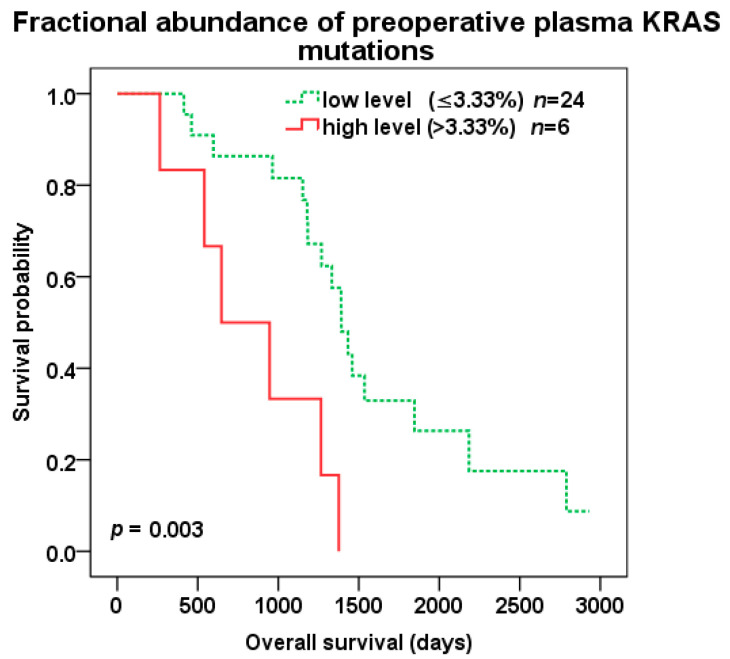
Overall survival (OS) after liver surgery for all patients with low (green color dotted line) vs. high (red color) KRAS fractional abundance in pre-operative plasma samples (cut-off 3.33%).

Patients with high compared to low level of preoperative KRAS pFA (cut-off 3.33%) experienced shorter OS (647 vs. 1392 days, *p* = 0.003; HR 4.391, 95%CI 1.529–12.614) (Figure 4), but not DFS (*p* = 0.118). Among 11 pKRAS+ patients, a high level of preoperative pFA (cut-off 3.33%) also predicted shorter OS (647 vs. 1459 days, *p* = 0.009; HR 10.733, 95%CI 1.236–93.24), but not DFS (*p* = 0.3).

In patients treated for CLM, a high level of preoperative KRAS pFA (cut-off 3.33%) remained an independent negative prognostic factor for OS after liver surgery also in multivariate analysis (Table 3).

**Figure 5:**
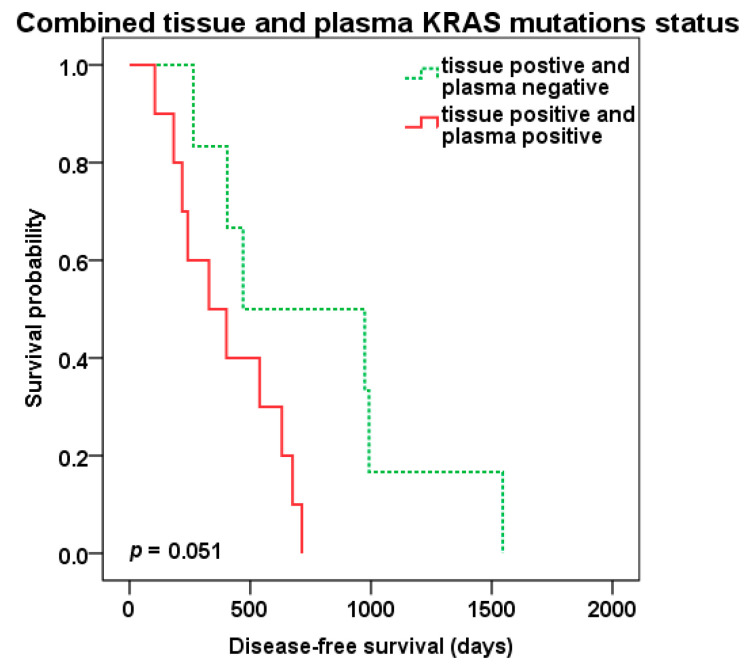
Disease-free survival (DFS) after liver surgery in patients with combined KRAS liver metastasis tissue and preoperative plasma positivity (red color) vs. tissue positivity only (green color dotted line).

Patients positive for KRAS mutations in both tissue of liver metastasis and preoperative plasma samples (tKRAS+/pKRAS+, *n* = 10) compared to patients with tissue KRAS positivity only (tKRAS+/pKRAS-, *n* = 6) showed shorter DFS (329 vs. 470 days, *p* = 0.051) (Figure 5), but not OS (*p* = 0.328).

**Figure 6:**
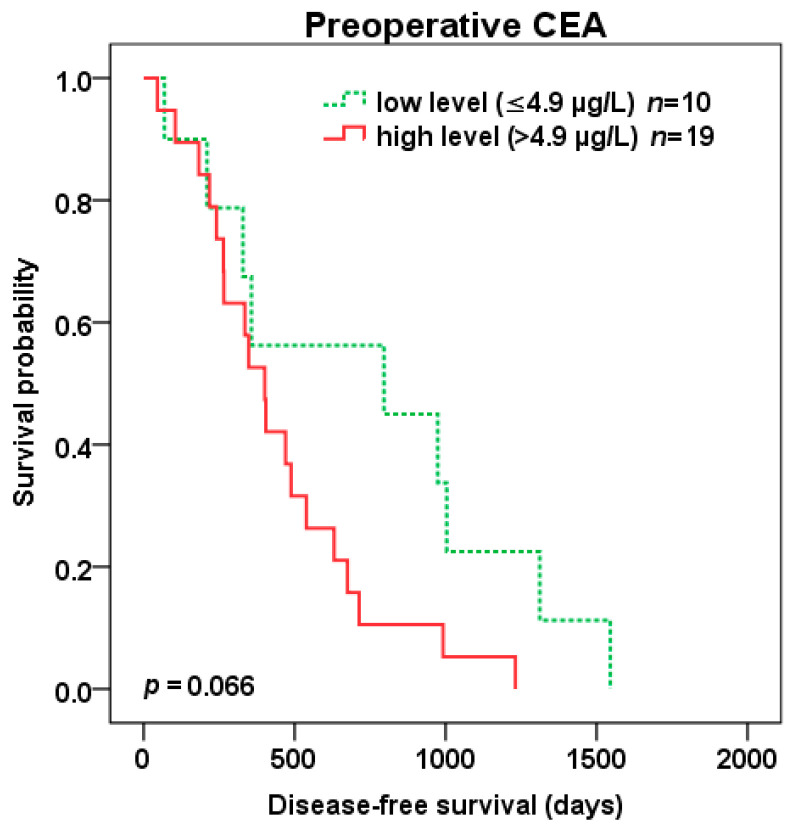
Disease-free survival (DFS) after liver surgery for patients with low (green color dotted line) vs. high (red color) preoperative CEA level (cut-off 4.9 µg/L).

**Figure 7:**
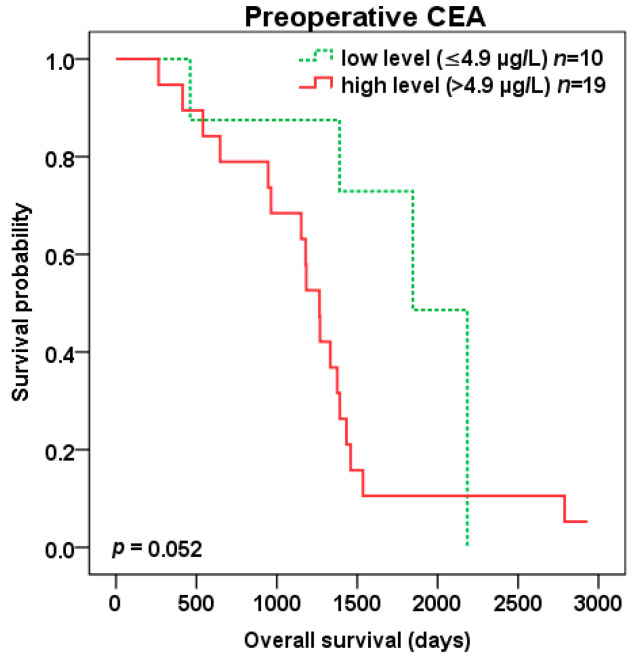
Overall survival (OS) after liver surgery for patients with low (green color dotted line) vs. high (red color) preoperative CEA level (cut-off 4.9 µg/L).

Patients with high compared to low preoperative CEA level (cut-off 4.9 µg/L) experienced trend to shorter DFS (401 vs. 796 days, *p* = 0.066) (Figure 6) as well as OS (1265 vs. 1846 days, *p* = 0.052) (Figure 7) after liver surgery.

**Figure 8:**
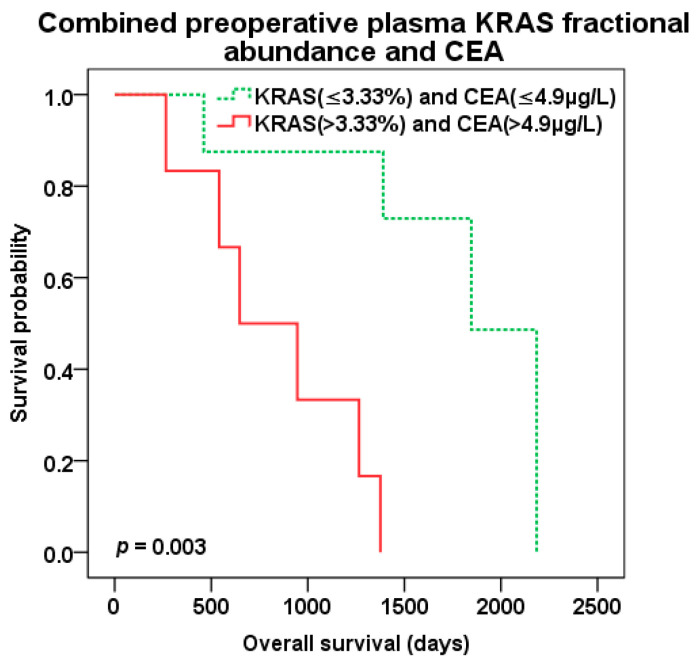
Overall survival (OS) after liver surgery for patients with combined low (green color dotted line) vs. high (red color) preoperative KRAS pFA and CEA levels (cut-off 3.33% and 4.9 µg/L, resp.).

**Figure 9:**
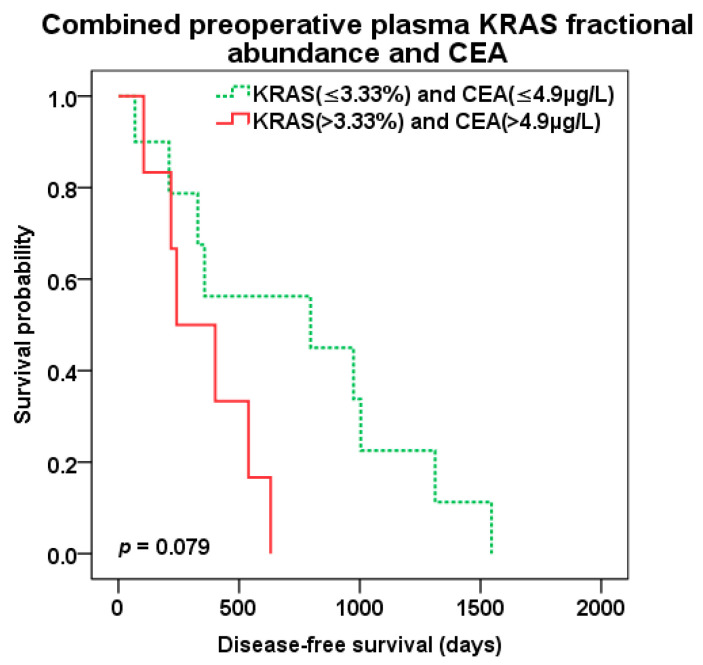
Disease-free survival (DFS) after liver surgery for patients with combined low (green color dotted line) vs. high (red color) preoperative KRAS pFA and CEA levels (cut-off 3.33% and 4.9 µg/L, resp.).

The best predictive value of patient survival after liver surgery was observed with combination of preoperative KRAS pFA and CEA level of CLM patients. Patients with both high pFA and CEA preoperative levels (cut-off 3.33% and 4.9 µg/L, resp.) experienced the worst survival compared to those with combined low pFA and CEA levels and the best OS (647 vs. 1846 days, *p* = 0.003; HR 13.638 95%CI 1.567–118.725) (Figure 8). There was trend to better DFS (*p* = 0.079) for combined low levels of KRAS pFA and CEA as well (Figure 9). Patients with both high levels of preoperative KRAS pFA and CEA showed also the worst OS (HR 44.877 95%CI 1.590–1266.479) in multivariate analysis including other covariates (extend of liver resection, presence of biological treatment).

## 3. Discussion

In the last two decades, we have witnessed an ever-increasing number of modalities of cancer treatment, including a growing spectrum of biological drugs and low molecular weight inhibitor drugs used for targeted treatment of cancer patients. At present, the correct choice of treatment depends also on the determination of prognostic and predictive biomarkers. The ability to respond in the case of recurrence/progression of the disease by changing cancer treatment carries with the need for real-time knowledge of the changes in the genotype of the tumor. This is where the liquid biopsy approach turns out to be very suitable for the patient, compensating in certain cases for the unavailability of the tumor tissue (biopsy, FFPE) and also allowing easy repeated peripheral blood sampling and the analysis of molecules with an assumed origin in the tumor tissue.

Moreover, the liquid biopsy approach overcomes to some extent the problem of tumor heterogeneity that complicates the traditional histopathological examinations. Hand in hand with the development of treatment modalities, more sensitive methods of detection of biomarkers (DNA mutations (ctDNA), oncoproteins and circulating tumor cells (CTC)) are becoming available, on the basis of which it is possible to determine prognosis or decide on an appropriate treatment. The decline in prices for individual determinations is also favorable, especially in the area of DNA analysis. The ddPCR method provides a sensitive quantitative determination of the genotypes of genes, enabling the determination of mutated DNA on the background of the wild-type majority.

This takes the possibilities of the liquid biopsy approach, specifically of the analysis of the mutated DNA released by the tumor tissue, a step further and allows not only the determination of low ctDNA levels but also the choice of a suitable cut-off for further categorization of cancer patients and a correct assessment of the relationship between the tumor tissue genotype and the phenotype, i.e., the character and extent of the oncological disease. It turns out that different biomarkers, in which a significant relationship to the clinicopathological characteristics of the tumor was found, do not characterize the same properties of the tumor. Therefore a combination of biomarkers based on different processes of the molecular biology of the tumor (protein, miRNA, DNA genotype) may be appropriate, especially for determining the prognosis. In this study, we decided to implement this approach.

The aim of the study was to determining concordance and prognostic significance of level of the KRAS mutations, to establish the possibility of using plasma levels of mutated KRAS ctDNA as a replacement for FFPE tissue for prediction of treatment in cases where FFPE tissue is not available. We used preoperative and postoperative plasma samples of patients treated for primary colorectal carcinoma and then it‘s liver metastases and the corresponding FFPE tissue samples together with long-term clinical data for determining of the prognostic significance of mutated KRAS level determined by the ddPCR method even in combination with routinely determined tumor marker CEA.

In patients with primary colorectal cancer, we detected KRAS mutation in the tissue in 54% of cases, in liver metastases of these patients it was 47.8% of cases. This corresponds to the upper limit given for larger sample sets [39]. The concordant mutation status between the primary carcinoma and liver metastasis was found in the majority of patients (93.4%). In three patients (9.375%) with primary cancer positivity, no mutation of the KRAS oncogene was observed in the metastasis, the opposite in one patient (7.69%).

It makes sense to discuss the concordance, due to the number of samples, between the presence of a mutation in the KRAS oncogene in liver metastasis tissue and the plasma of a preoperative sample of liver metastases only. The overall percentage agreement between plasma-based and tissue-based KRAS mutation status was 75.9%. Several previous studies evaluated the concordance of KRAS alterations between tissue and ctDNA and found overall concordance to range 67–96% [40]. We also evaluated the relationship between the levels of KRAS mutated fraction of ctDNA and the levels of determined mutations in liver metastasis tissue. The KRAS fraction abundance positively correlated (R = 0.649) between preoperative plasma samples and tissue samples of liver metastases. These results indicate the limits of liquid biopsy as a substitute to tissue biopsy KRAS mutations analysis for treatment prediction. At the same time, this indicate that the results of plasma vs. tissue assays provide different insights into the biology of tumor behavior, discussed below. KRAS mutation in plasma disappeared after removal of metastases in all patients where the assessment was available. It is possible due to relatively short half-life time of DNA in plasma ranging from 15 min to several hours. It also points to the possibility of minimal residual disease detection and non-invasive monitoring if tumor mass was completely removed by surgery in patients with mutated KRAS. Likewise, achieving zero KRAS ctDNA level after liver surgery is important for early detection of potential disease recurrence, where the KRAS ctDNA can increase again.

We also investigated the relationship of KRAS mutated DNA levels to DFS and OS, both tissue and plasma levels. Based on the concordance of the presence of KRAS mutations in the plasma and tissue of patients with liver metastases, we would expect similar results regarding the possible prognostic significance (OS and DFS). However, we recorded prognostic significance only for the determination of mutated DNA of the KRAS gene in plasma. Patients with a fractional abundance value lower than 3.3% had got a significantly better prognosis (OS). Based on the relationship between the level of KRAS mutated ctDNA and prognosis, our results indicate that, in certain cases, it may be more appropriate to determine the presence of a KRAS mutation based on plasma results.

Other studies showed that the quantification of ctDNA is the valuable prognostic factor for CLM patients and support the idea of non-invasive detection of persistent minimal residual disease. In the study by Cassinotti et al. and Frattini et al., the ctDNA concentration significantly decreased after primary tumor resection. However, the ctDNA concentration dramatically increased in patients with a relapse. In “disease-free” patients, the ctDNA level remains in decreasing tendency [41,42]. The preoperative assessment of ctDNA level might be useful for better prognosis estimation and a postoperative assessment could early detect the cancer recurrence. Increased ctDNA level significantly correlated with an unfavorable prognosis in another study as well [43]. In addition, our study showed the benefits of quantifying of copies of KRAS ctDNA by the ddPCR.

In our opinion based on these results, it can be stated that the plasma level of the KRAS mutation does not reflect the same biological properties of oncological disease as the level of the mutated KRAS oncogene DNA determined in the tumor tissue. Plasma assays also include information on the ability to release tumor molecules into the bloodstream. In addition, the result shows that we do not have two groups of patients (KRAS+ and KRAS-), but rather three groups. The group without a mutation in the KRAS oncogene, with a small amount of ctDNA in the bloodstream and a large amount of ctDNA.

There is evidence that ctDNA assessment could be a more preferable biomarker of prognosis than CEA plasma levels. Vymetalkova et al. mentions that, at the time of recurrence, 80% of CRC patients were ctDNA positive, while CEA levels were only elevated in 41% of CRC patients [43]. Reinert et al. [44] and Carpinetti et al. [45] published that ctDNA assessment in follow-up period of CRC patients may show cancer recurrence and therapy response an earlier in comparison with monitoring of CEA or radiological evaluation. We observed the benefit of combining both ctDNA and CEA levels for prognosis.

The limitations of our study must also be mentioned. These are mainly heterogenous treatment protocols applied for oncological patients and also the limited number of plasma samples available for analysis. On the other hand, the strengths are the use of the ddPCR method and detailed long-term patient follow-up with a high maturity of the cohort of patients. The assay for ddPCR used in our study is able to screen spectrum of KRAS mutations (as described in materials and methods) that is very useful in the case of ctDNA analysis and limited amount of isolated DNA. This advantage is limited by the impossibility to discriminate among individual mutations.

## 4. Materials and Methods

### 4.1. Patients and Samples

We retrospectively analyzed 71 patients who underwent primary surgery for colorectal carcinoma and after the onset of disease progression into the liver who underwent surgery for CRC liver metastases (Table 4). The study was approved by the institutional review board and local ethics committee of the University Hospital in Pilsen. The ethical code is no.1552016. Every patient signed an informed consent form for the use of their blood samples in clinical research for the assessment of tumor markers. Each FFPE sample of CRC and CLM patients was verified by a pathologist diagnosis. All CRC tumors used in these study were histologically adenocarcinomas. CRC patients were staged base on the TNM system of the International Union Against Cancer (IUCC, 7th edition) [46]. The median patients´ follow-up was 4.1 years. The evaluation of remission and recurrence were classified based on response evaluation criteria in solid tumors (RECIST) [47].

The quantitative estimation of 7 KRAS G12/G13 mutations was performed by ddPCR in 71 colorectal carcinoma tissue samples (FFPE) and paired 71 liver metastasis tissue samples (FFPE). In these patients the quantitative KRAS estimation was also done in 7 preoperative (taken a day before primary CRC surgery) and postoperative (taken a day after primary CRC surgery) plasma samples. Similarly the mutated KRAS was quantified in 30 preoperative and postoperative plasma samples of patients undergoing surgery for CLM.

### 4.2. Blood Samples

The peripheral blood samples were collected from the cubital vein using K3EDTA Vacutainer tubes (Greiner Bio-One, Kremsmünster, Austria). Plasma was prepared by two-step centrifugation of 6 mL of blood at 950 rcf at 10 min at 4 °C and then at 11,000 rcf at 10 min at 4 °C–to remove any cell debris as a standard procedures used for cell-free DNA assessment.

For CEA detection, the blood serum was separated by centrifugation at 1700 rcf for 10 min from 4 mL of blood collected in Vacuette^®^ blood collection tubes (Greiner Bio-One, Kremsmünster, Austria). Until analysis plasma and serum samples were stored frozen at −80 °C.

### 4.3. DNA Isolation

Tissue DNA was isolated by AllPrep DNA/RNA FFPE kit (Qiagen, Hilden, Germany). Total circulating DNA was isolated from 1 mL plasma by the QIAamp Circulating Nucleic Acid Kit kit (Qiagen, Hilden, Germany), according to the manufacturer manual.

### 4.4. ddPCR Assay

Quantitative estimation of 7 KRAS G12/G13 mutations was done by ddPCR™ KRAS G12/G13 Multiplex Screening Kit (catalogue No. 186-350, Bio-Rad Laboratories, Hercules, CA, USA) on QX200 Droplet Digital PCR System (Bio-Rad Laboratories, Hercules, CA). This assay enables to screen KRAS variants G12A (dHsaCP2500586), G12C (dHsaCP2500584), G12D (dHsaCP2500596), G12R (dHsaCP2500590), G12S (dHsaCP2500588), G12V (dHsaCP2500592) and G13D (dHsaCP2500598).

For FFPE samples the total DNA load in reaction was 50 ng of isolated DNA per well. To overstep the small amounts of mutated DNA in circulation generally, all plasma samples were analyzed in 3 well simultaneously in each 5 µL of isolated DNA without dilution was added yielded 25–225 ng of DNA per 3 merged wells. Data obtained in 3 wells (same sample) were merged for results as recommended in Bulletin 6628A, Rare mutation detection (Best practices guidelines) by Bio-Rad Laboratories, Hercules, CA, USA.

The analytical procedure was performed according to PrimePCR ddPCR assay manual-Bio-Rad 10,048,179 Rev. A, (Bio-Rad Laboratories, Hercules, CA, USA). No DNA digestion was used for samples, while for FFPE and cell free DNA samples is not required. PCR master mix was prepared by 11 µL 2× ddPCR Supermix for Probe (no dUTP), 1 µL multiplex primers/probes (FAM + HEX) and 7.5 µL nuclease-free water for each FFPE sample. For cell free DNA sample was 33 µL 2× ddPCR Supermix for Probe (no dUTP), 3 µL multiplex primers/probes (FAM + HEX) and 15 µL nuclease-free water for three wells. There was manually transferred 20 µL of final PCR mix into wells of a CG 8 cartridge and added 70 µL Droplet Generation Oil for probes. Cartridge is loaded into QX200TM Droplet Generator (Bio-Rad Laboratories, Hercules, CA, USA) for droplet generation, followed by transfer of 40 µL of sample droplets into the 96-well PCR plate. Plate is heat sealed by foil using PX1 PCR Plate Sealer (Bio-Rad Laboratories, Hercules, CA, USA). Amplification was performed in T100TM Thermal Cycler with 96-Deep Well Reaction Module (thermal cycling protocol: 95 °C for 10 min, 40× cycle of 94 °C for 30 s, 55 °C for 1 min, 98 °C for 10 min (all ramp rate 2 °C/s), and final cooling down to 4 °C, ramp rate 1 °C/s). After thermal cycling, reading analysis was performed on QX200TM Droplet Reader (Bio-Rad Laboratories, Hercules, CA, USA) with setting for FAM/HEX.

In all plates, positive controls for each detected KRAS mutation and negative samples were screened for quality control and “cut-off” set-up. Control samples have checked DNA quality by amplification of control genes: 100–600bp (BIOMED-2), detection of mutations in positive control samples were performed by PCR and reverse-hybridization accredited according to CSN EN 15189:2013.

Data were analyzed in Biorad-Laboratories software Quantasoft 1.7 and QuantaSoft™ Analysis Pro 1.0. Thresholds were placed manually. Fractional abundance (FA) of targeted mutations in samples, determined as positive, were calculated as percentage FA% = mutated copies/(mutated copies + wildtype copies) × 100. FFPE samples, for which positive droplet count (FAM plus HEX channel) was insufficient, were reanalyzed with DNA load app. 100 ng/well reaction. 

According to false positive rate (FPR) 1625 (cumulatively 13 false positive droplets in 8 control samples) in plasma sample plates, the 5 positive droplets were determined as a cut-off for merged plasma sample results.

To avoid any false positive results for FFPE samples, we have chosen relatively high cut-off value: the fractional abundance of 5% for positivity of mutation occurrence, while mean FA% of negative controls was 1.5% and maximum FA% in FFPE negative controls was approximately 3% in FFPE plates.

### 4.5. Quantitative Measurement of CEA

Serum levels of CEA protein were determined in monoplicates by chemiluminescent assay the ACCESS CEA assay with the UniCel DxI 800 Instrument (Beckman Coulter, Brea, CA, USA). The cut off value of the preoperative serum CEA level was set at 4.9 ng/mL.

### 4.6. Statistical Analysis

Statistical analysis was performed using the SPSS 22 software package (IBM, Armonk, NY, USA). The statistical significance was considered with a *p* value of ≤0.05. Kaplan–Meier method with an “optimal cut off” found with the lowest *p*-value of the log rank test was used for survival analysis. Multivariate analysis was performed by the Cox regression model to test independent significance while adjusting for covariates. Data were presented as hazard ratios (HR) and 95% confidence intervals (95%CI). Spearman’s rank-order correlation method was used for mutual correlations. The Wilcoxon signed-rank test was used for comparison of differences between pre- and post-operative levels of circulating markers.

## 5. Conclusions

Published data on liquid biopsy based on ctDNA in colorectal cancer patients with advanced disease provide additional information on the course and prognosis of the disease and may help with the therapy decision making process. The best prognostic value in colorectal cancer patients undergoing surgery for liver metastases was observed based on the detection of the level of KRAS ctDNA in combination with routinely determined tumor marker CEA.

## Figures and Tables

**Table 1:** The concordance between tissue KRAS status (KRAS positive vs. KRAS negative) in primary tumor (CRC) and liver metastases (CLM).

Tissue Origin		CRC	
	KRAS Status	KRAS Negative	KRAS Positive
**CLM**	**KRAS negative**	28	3
	**KRAS positive**	1	29

**Table 2:** The concordance between tissue KRAS status (KRAS positive vs. KRAS negative) in colorectal cancer liver metastases (CLM) and preoperative plasma samples (Plasma).

Tissue vs. Plasma Origin		CLM Tissue	
	KRAS Status	KRAS Negative	KRAS Positive
**Plasma**	**KRAS negative**	12	6
	**KRAS positive**	1	10

**Table 3:** The multivariate analysis of disease free survival (DFS) and overall survival (OS) of patients undergoing liver surgery.

The Multivariate Survival Analysis	DFS		OS	
Status of Biomarkers and Clinical Characteristics	HR (95% Confidence Interval)	Significance	HR (95% Confidence Interval)	Significance
**Plasma KRAS fractional abundance [cut-off 3.33%]**				
Low level	1.000		1.000	
High level	2.542 (0.823–7.853)	*p* = 0.105	11.732 (2.729–50.432)	***p*** **= 0.001**
**CEA [cut-off 4.9 µg/L]**				
Low level	1.000		1.000	
High level	3.264 (1.119–9.521)	***p*** **= 0.03**	5.409 (1.272–22.998)	***p*** **= 0.022**
**Extend of liver resection**				
R0	1.000		1.000	
R1	3.309 (1.09–10.045)	***p*** **= 0.035**	6.054 (1.499–24.451)	***p*** **= 0.011**
RFA ^1^	2.895 (0.891–9.406)	*p* = 0.077	7.368 (1.479–36.7)	***p*** **= 0.015**
**Biological treatment**				
bevacizumab or cetuximab	1.000		1.000	
n.a.	1.546 (0.633–3.774)	*p* = 0.339	0.828 (0.326–2.104)	*p* = 0.692

^1^ Radiofrequency ablation.

**Table 4:** Patient characteristics.

Patient Characteristics	Number of Patients (%)
**All patients in the study**	71 (100%)
**Gender**	
Female	29 (41%)
Male	42 (59%)
**Mean age at liver surgery (min-max)**	62.7 (29–77) years
**Extent of liver surgery**	
R0 resection	38 (54%)
R1 resection	21 (30%)
RFA ^1^	11 (16%)
**Adjuvant chemotherapy**	
Yes	55 (77%)
No	16 (23%)
**Adjuvant biological therapy (bevacizumab or cetuximab)**	
Yes	15 (21%)
No	56 (79%)
**Tumor grade (available in 44 pts.)**	
Grade 1	15 (21%)
Grade 2	23 (32%)
Grade 3	6 (9%)
**Number of liver metastases**	
1	35 (49%)
≥2	36 (51%)
**Mean size of metastases (min-max)**	38.5 (6–130) mm
**Extrahepatic disease**	
Yes	4 (6%)
No	67 (94%)

^1^ Radiofrequency ablation.

## Abbreviations

95% CI	95% Confidence interval
CEA	Carcinoembryonic antigen
CLM	Colorectal cancer liver metastases
CRC	Colorectal cancer
CTC	Circulating tumor cells
cfDNA	Circulating cell-free deoxyribonucleic acid
ctDNA	Circulating cell-free tumor deoxyribonucleic acid
ddPCR	Droplet digital polymerase chain reaction
DFS	Disease-free survival
DMFS	Distant metastasis free survival
DNA	Deoxyribonucleic acid
EGFR	Epidermal growth factor receptor
FFPE	Formalin-fixed, paraffin-embedded
FPR	False positive rate
HR	Hazard ratio
IUCC	International union against cancer
KRAS	Kirsten rat sarcoma viral oncogene homolog
mAb	Monoclonal antibody
miRNA	Micro-ribonucleic acid
NRAS	Neuroblastoma RAS viral oncogene homolog
OS	Overall survival
PCR	Polymerase chain reaction
pFA	Plasma KRAS fractional abundance
pKRAS-	Plasma KRAS negativity
pKRAS+	Plasma KRAS positivity
RECIST	Response evaluation criteria in solid tumors
RFA	Radiofrequency ablation
tFA	Tissue KRAS fractional abundance
tKRAS+	Tissue KRAS positivity
TNM	Tumor, node, metastases
